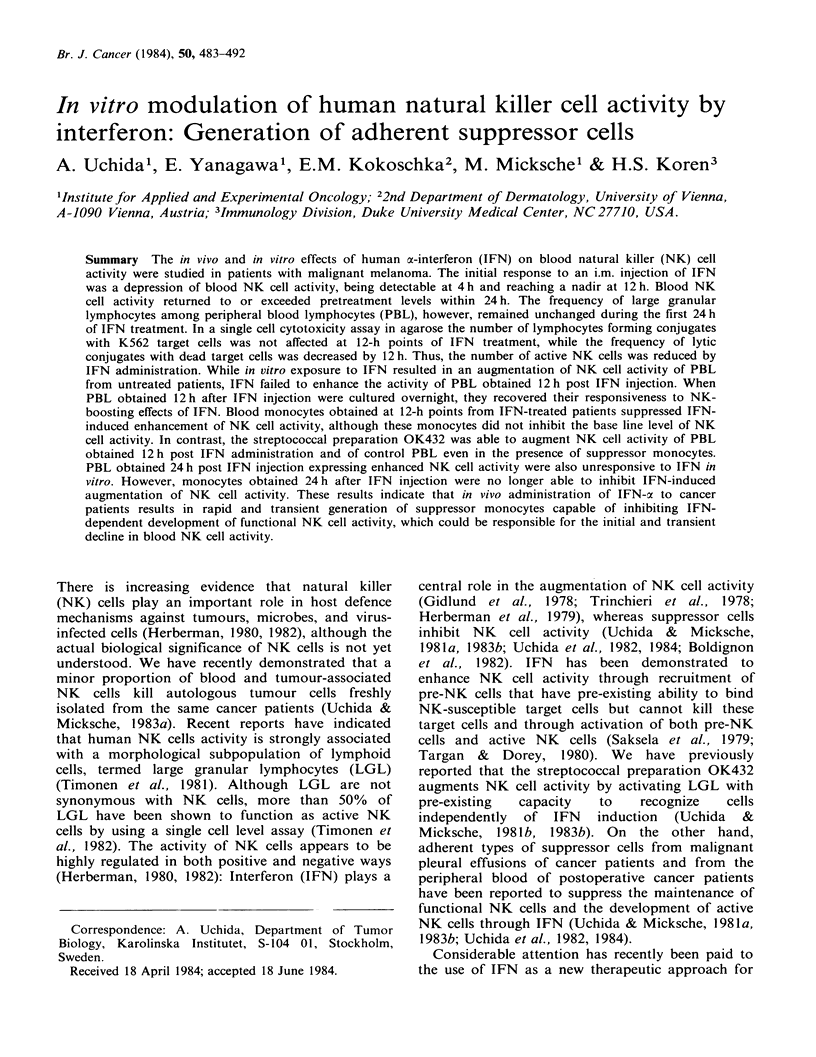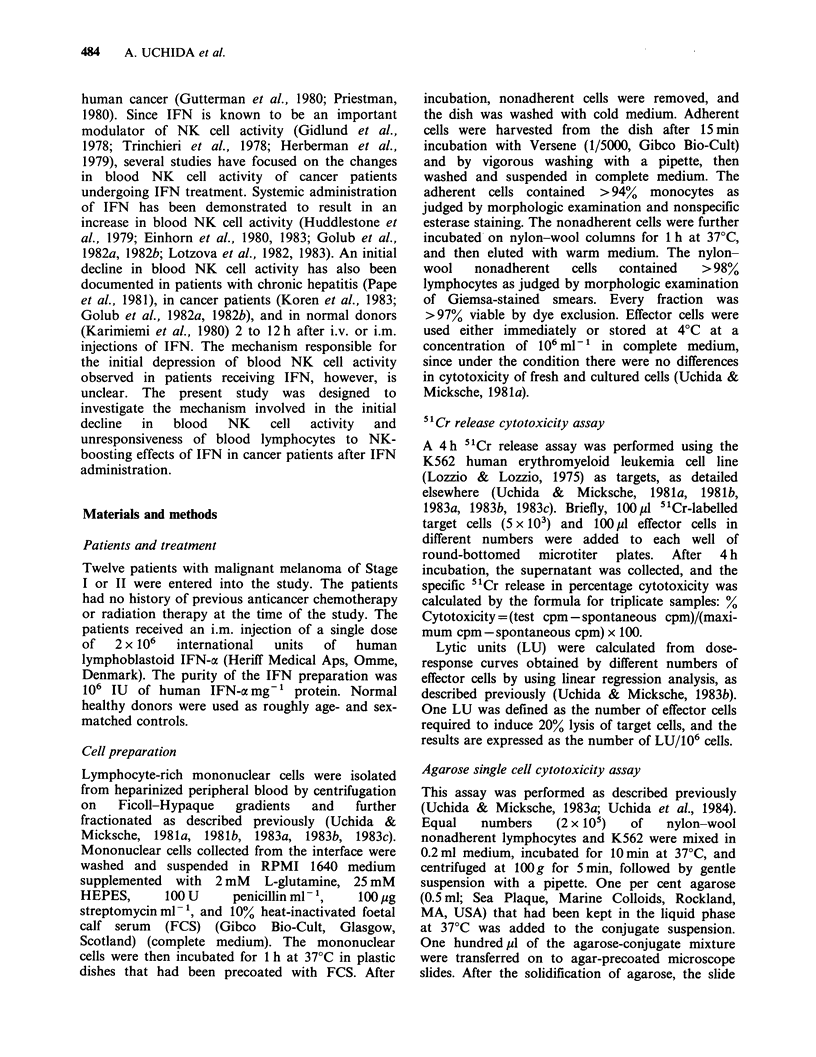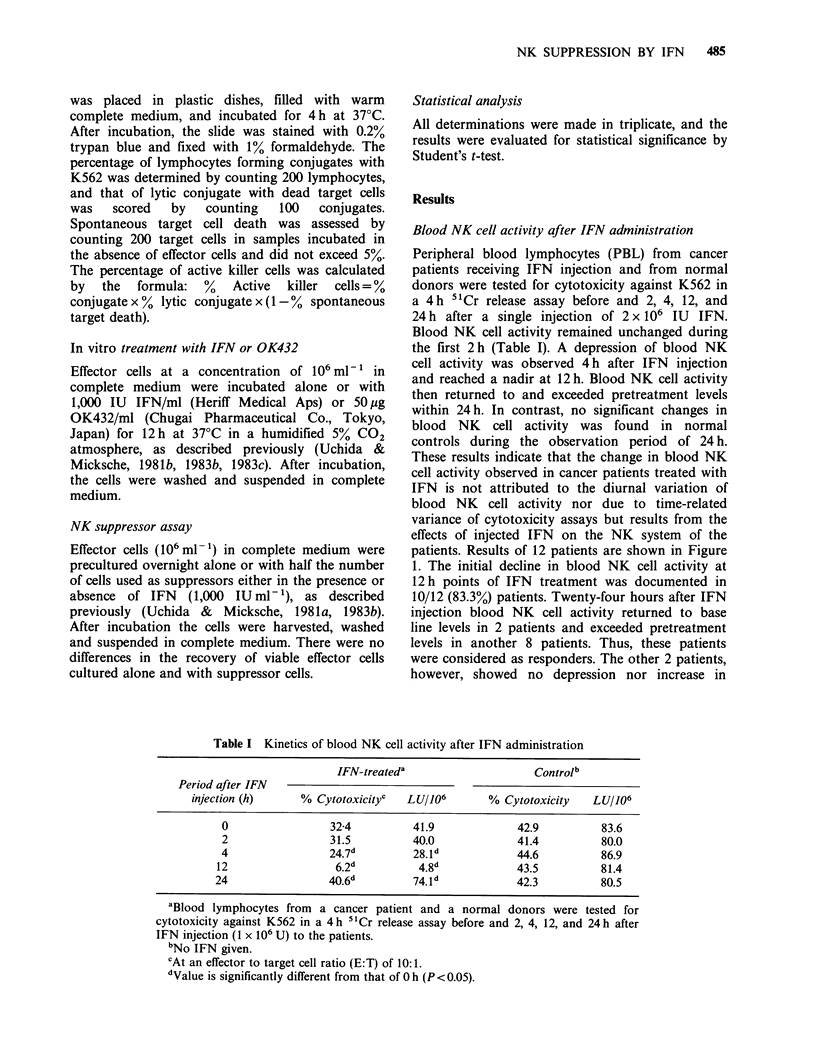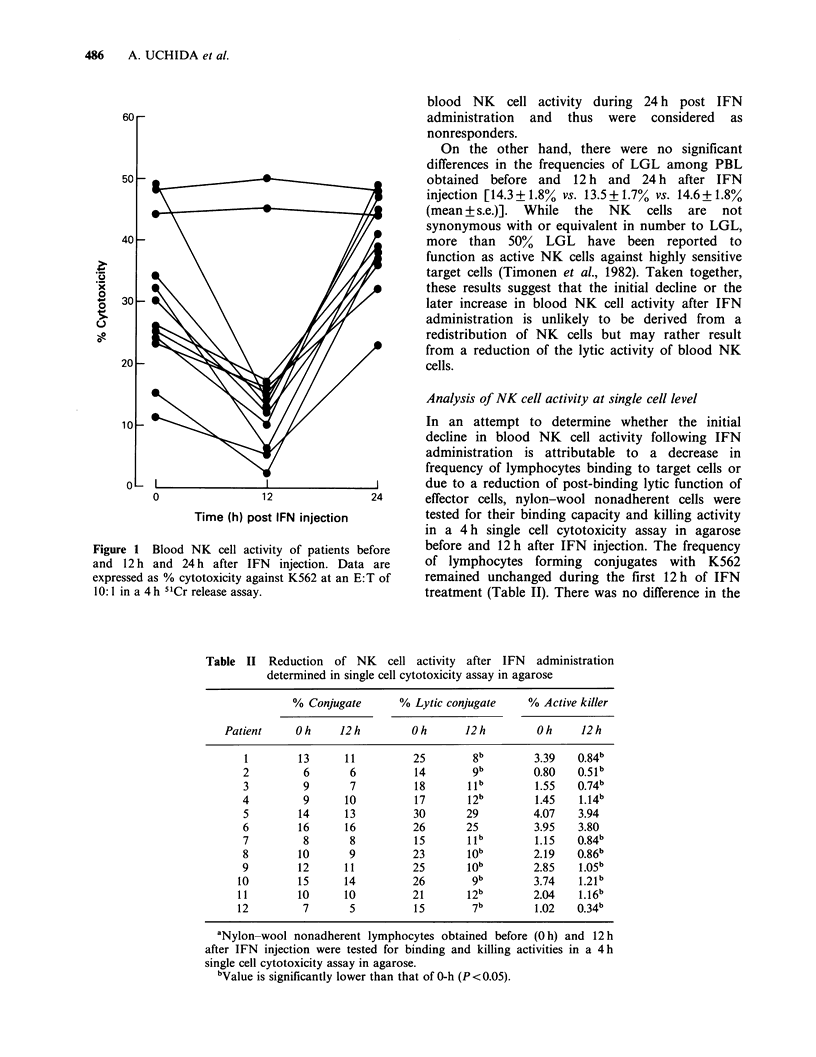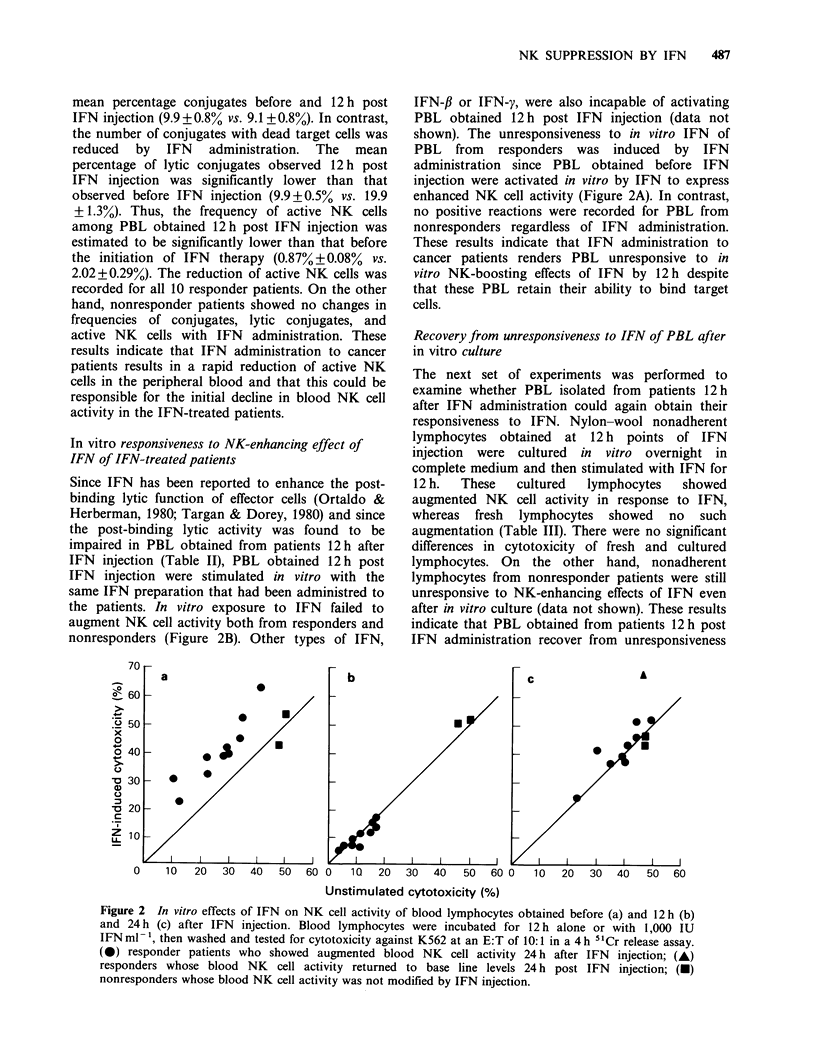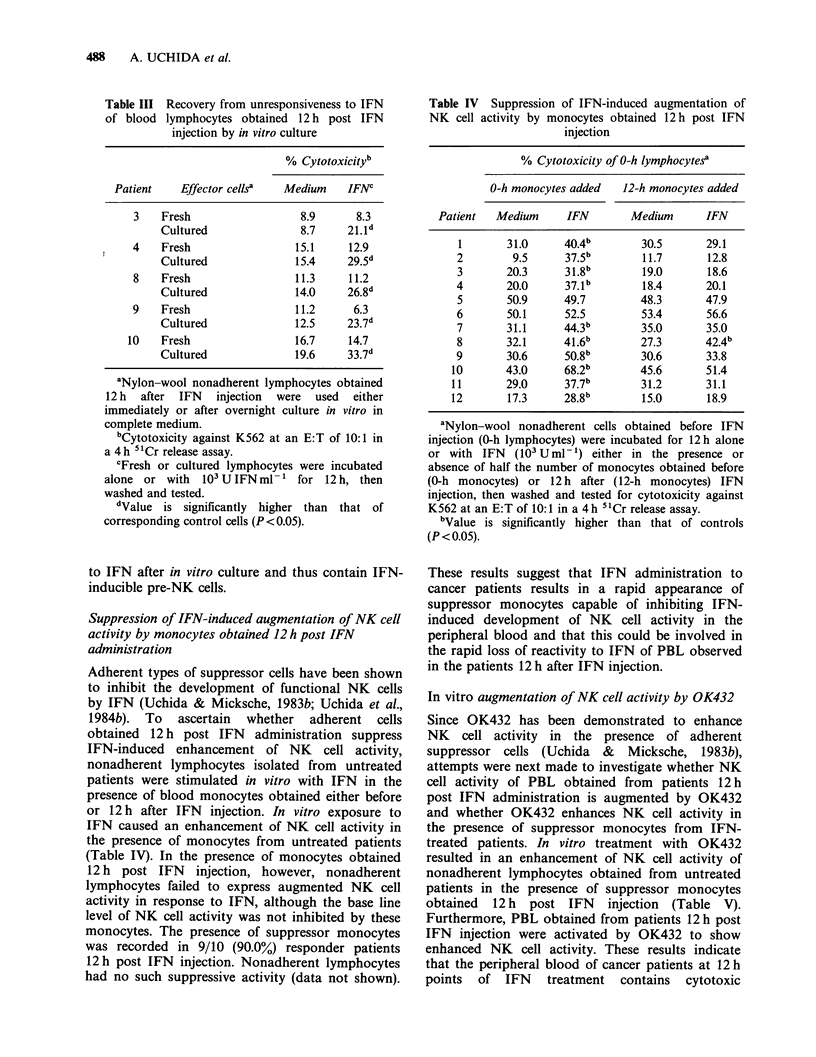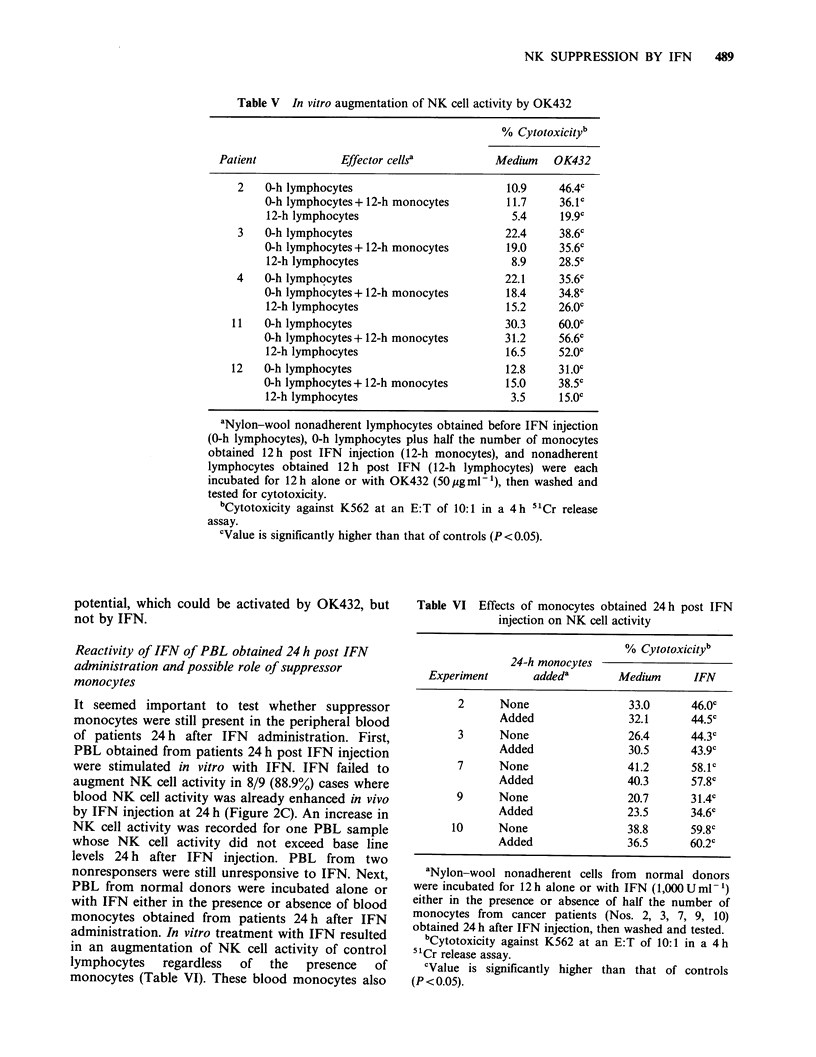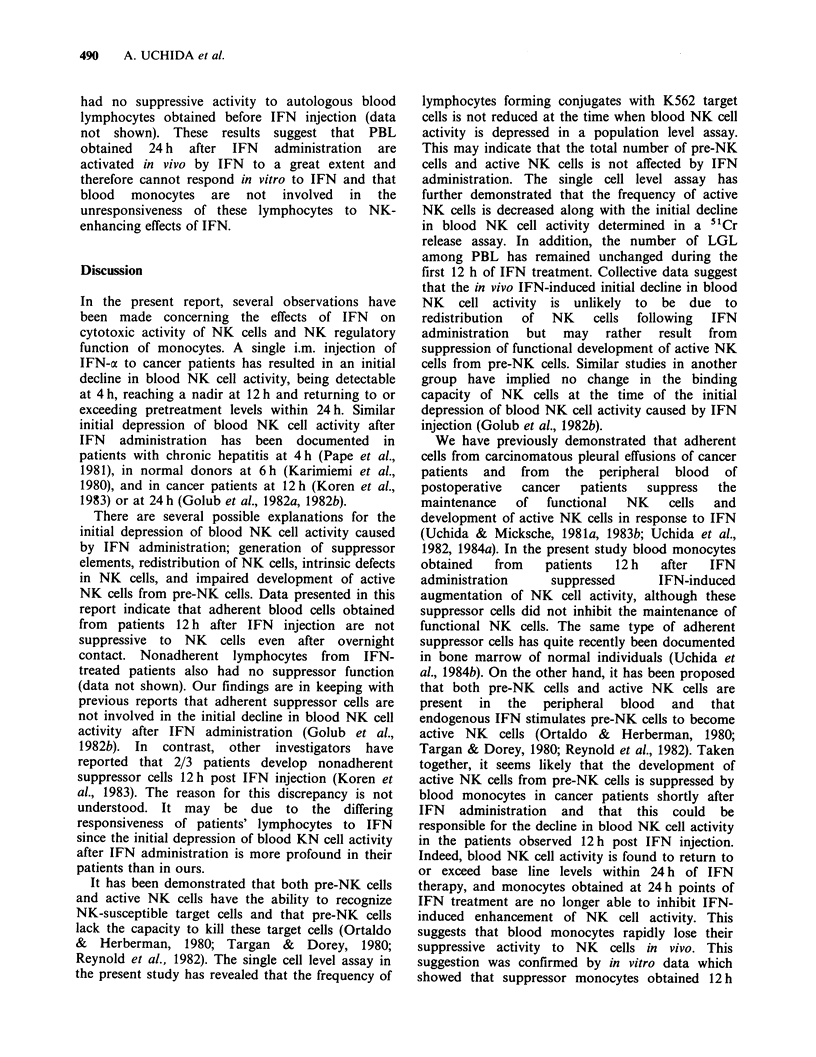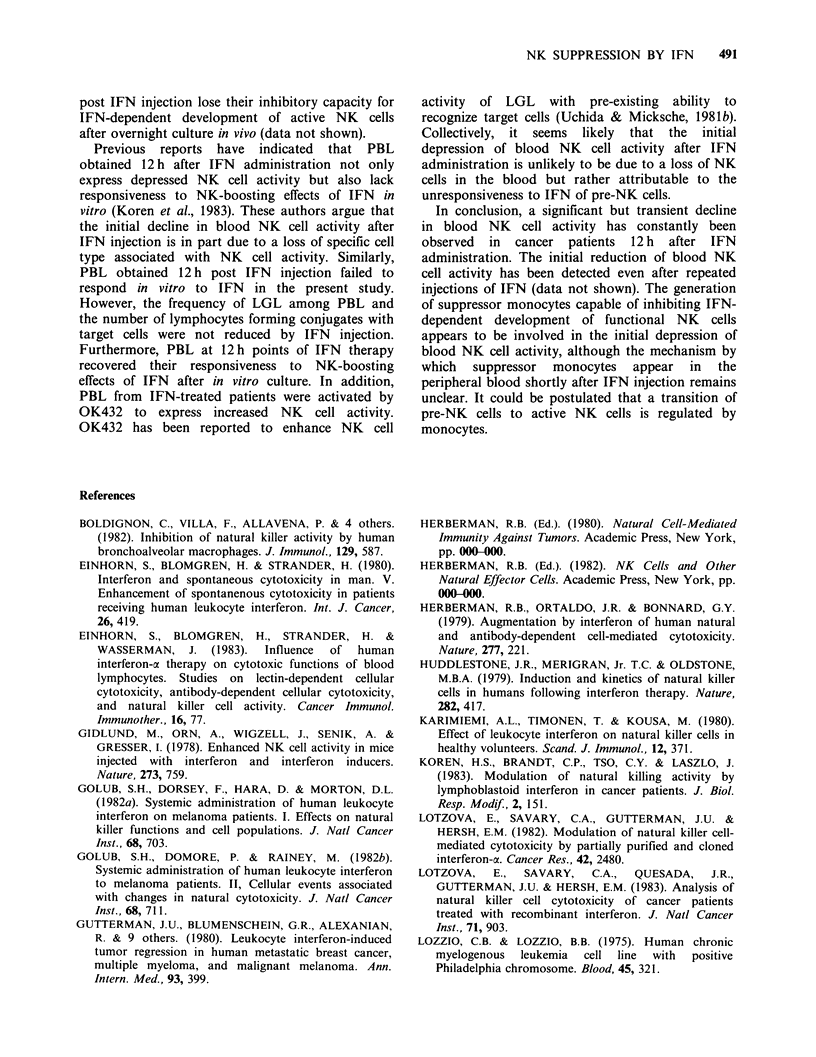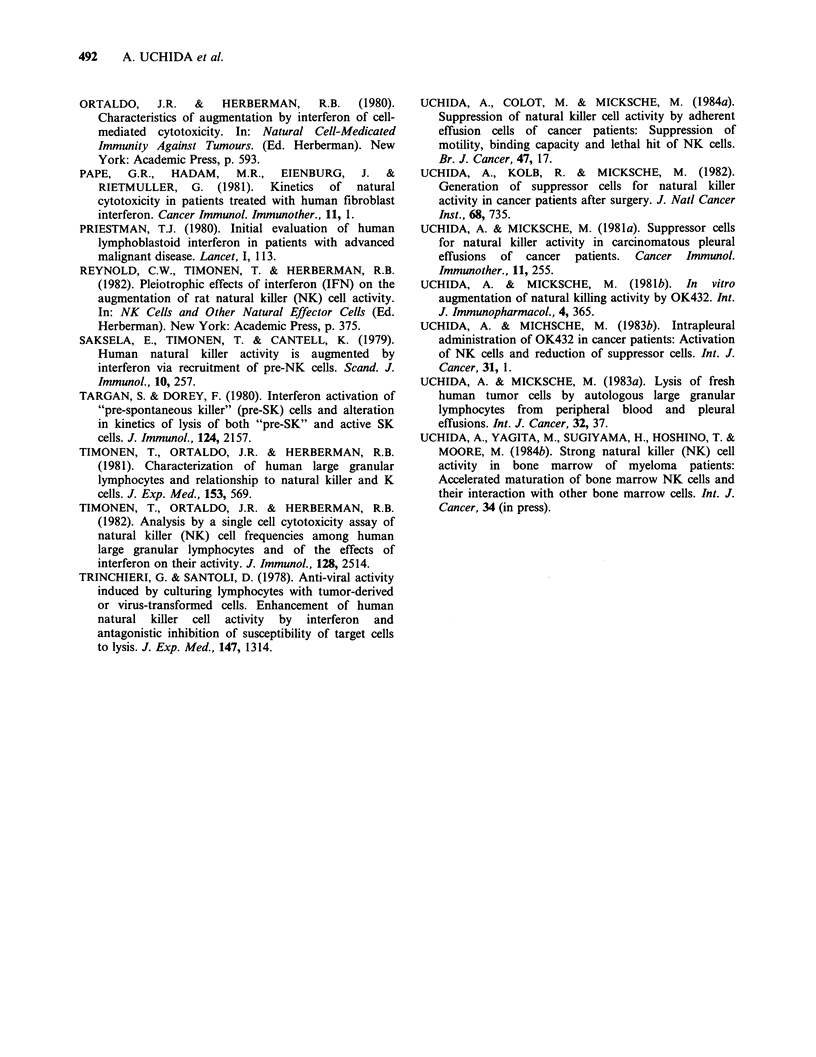# In vitro modulation of human natural killer cell activity by interferon: generation of adherent suppressor cells.

**DOI:** 10.1038/bjc.1984.205

**Published:** 1984-10

**Authors:** A. Uchida, E. Yanagawa, E. M. Kokoschka, M. Micksche, H. S. Koren

## Abstract

The in vivo and in vitro effects of human alpha-interferon (IFN) on blood natural killer (NK) cell activity were studied in patients with malignant melanoma. The initial response to an i.m. injection of IFN was a depression of blood NK cell activity, being detectable at 4 h and reaching a nadir at 12 h. Blood NK cell activity returned to or exceeded pretreatment levels within 24 h. The frequency of large granular lymphocytes among peripheral blood lymphocytes (PBL), however, remained unchanged during the first 24 h of IFN treatment. In a single cell cytotoxicity assay in agarose the number of lymphocytes forming conjugates with K562 target cells was not affected at 12-h points of IFN treatment, while the frequency of lytic conjugates with dead target cells was decreased by 12 h. Thus, the number of active NK cells was reduced by IFN administration. While in vitro exposure to IFN resulted in an augmentation of NK cell activity of PBL from untreated patients, IFN failed to enhance the activity of PBL obtained 12 h post IFN injection. When PBL obtained 12 h after IFN injection were cultured overnight, they recovered their responsiveness to NK-boosting effects of IFN. Blood monocytes obtained at 12-h points from IFN-treated patients suppressed IFN-induced enhancement of NK cell activity, although these monocytes did not inhibit the base line level of NK cell activity. In contrast, the streptococcal preparation OK432 was able to augment NK cell activity of PBL obtained 12 h post IFN administration and of control PBL even in the presence of suppressor monocytes. PBL obtained 24 h post IFN injection expressing enhanced NK cell activity were also unresponsive to IFN in vitro. However, monocytes obtained 24 h after IFN injection were no longer able to inhibit IFN-induced augmentation of NK cell activity. These results indicate that in vivo administration of IFN-alpha to cancer patients results in rapid and transient generation of suppressor monocytes capable of inhibiting IFN-dependent development of functional NK cell activity, which could be responsible for the initial and transient decline in blood NK cell activity.


Br. J. Cancer (1984), **50**, 483–492

### *In vitro* modulation of human natural killer cell activity by interferon: Generation of adherent suppressor cells

A. Uchida[1], E. Yanagawa[1], E.M. Kokoschka[2], M. Micksche[1] & H.S. Koren[3]

[1]*Institute for Applied and Experimental Oncology;* [2]*2nd Department of Dermatology, University of Vienna, A-1090 Vienna, Austria;* [3]*Immunology Division, Duke University Medical Center, NC 27710, USA.*

**Summary** The *in vivo* and *in vitro* effects of human α-interferon (IFN) on blood natural killer (NK) cell activity were studied in patients with malignant melanoma. The initial response to an i.m. injection of IFN was a depression of blood NK cell activity, being detectable at 4 h and reaching a nadir at 12 h. Blood NK cell activity returned to or exceeded pretreatment levels within 24 h. The frequency of large granular lymphocytes among peripheral blood lymphocytes (PBL), however, remained unchanged during the first 24 h of IFN treatment. In a single cell cytotoxicity assay in agarose the number of lymphocytes forming conjugates with K562 target cells was not affected at 12-h points of IFN treatment, while the frequency of lytic conjugates with dead target cells was decreased by 12 h. Thus, the number of active NK cells was reduced by IFN administration. While *in vitro* exposure to IFN resulted in an augmentation of NK cell activity of PBL from untreated patients, IFN failed to enhance the activity of PBL obtained 12 h post IFN injection. When PBL obtained 12 h after IFN injection were cultured overnight, they recovered their responsiveness to NK-boosting effects of IFN. Blood monocytes obtained at 12-h points from IFN-treated patients suppressed IFN-induced enhancement of NK cell activity, although these monocytes did not inhibit the base line level of NK cell activity. In contrast, the streptococcal preparation OK432 was able to augment NK cell activity of PBL obtained 12 h post IFN administration and of control PBL even in the presence of suppressor monocytes. PBL obtained 24 h post IFN injection expressing enhanced NK cell activity were also unresponsive to IFN *in vitro*. However, monocytes obtained 24 h after IFN injection were no longer able to inhibit IFN-induced augmentation of NK cell activity. These results indicate that *in vivo* administration of IFN-α to cancer patients results in rapid and transient generation of suppressor monocytes capable of inhibiting IFN-dependent development of functional NK cell activity, which could be responsible for the initial and transient decline in blood NK cell activity.

There is increasing evidence that natural killer (NK) cells play an important role in host defence mechanisms against tumours, microbes, and virus-infected cells (Herberman, 1980, 1982), although the actual biological significance of NK cells is not yet understood. We have recently demonstrated that a minor proportion of blood and tumour-associated NK cells kill autologous tumour cells freshly isolated from the same cancer patients (Uchida & Micksche, 1983*a*). Recent reports have indicated that human NK cells activity is strongly associated with a morphological subpopulation of lymphoid cells, termed large granular lymphocytes (LGL) (Timonen *et al.*, 1981). Although LGL are not synonymous with NK cells, more than 50% of LGL have been shown to function as active NK cells by using a single cell level assay (Timonen *et al.*, 1982). The activity of NK cells appears to be highly regulated in both positive and negative ways (Herberman, 1980, 1982): Interferon (IFN) plays a central role in the augmentation of NK cell activity (Gidlund *et al.*, 1978; Trinchieri *et al.*, 1978; Herberman *et al.*, 1979), whereas suppressor cells inhibit NK cell activity (Uchida & Micksche, 1981*a*, 1983*b*; Uchida *et al.*, 1982, 1984; Boldignon *et al.*, 1982). IFN has been demonstrated to enhance NK cell activity through recruitment of pre-NK cells that have pre-existing ability to bind NK-susceptible target cells but cannot kill these target cells and through activation of both pre-NK cells and active NK cells (Saksela *et al.*, 1979; Targan & Dorey, 1980). We have previously reported that the streptococcal preparation OK432 augments NK cell activity by activating LGL with pre-existing capacity to recognize cells independently of IFN induction (Uchida & Micksche, 1981*b*, 1983*b*). On the other hand, adherent types of suppressor cells from malignant pleural effusions of cancer patients and from the peripheral blood of postoperative cancer patients have been reported to suppress the maintenance of functional NK cells and the development of active NK cells through IFN (Uchida & Micksche, 1981*a*, 1983*b*; Uchida *et al.*, 1982, 1984).

Considerable attention has recently been paid to the use of IFN as a new therapeutic approach for

Correspondence: A. Uchida, Department of Tumor Biology, Karolinska Institutet, S-104 01, Stockholm, Sweden.

Received 18 April 1984; accepted 18 June 1984.

human cancer (Gutterman et al., 1980; Priestman, 1980). Since IFN is known to be an important modulator of NK cell activity (Gidlund et al., 1978; Trinchieri et al., 1978; Herberman et al., 1979), several studies have focused on the changes in blood NK cell activity of cancer patients undergoing IFN treatment. Systemic administration of IFN has been demonstrated to result in an increase in blood NK cell activity (Huddlestone et al., 1979; Einhorn et al., 1980, 1983; Golub et al., 1982a, 1982b; Lotzova et al., 1982, 1983). An initial decline in blood NK cell activity has also been documented in patients with chronic hepatitis (Pape et al., 1981), in cancer patients (Koren et al., 1983; Golub et al., 1982a, 1982b), and in normal donors (Karimiemi et al., 1980) 2 to 12 h after i.v. or i.m. injections of IFN. The mechanism responsible for the initial depression of blood NK cell activity observed in patients receiving IFN, however, is unclear. The present study was designed to investigate the mechanism involved in the initial decline in blood NK cell activity and unresponsiveness of blood lymphocytes to NK-boosting effects of IFN in cancer patients after IFN administration.

### Materials and methods

### Patients and treatment

Twelve patients with malignant melanoma of Stage I or II were entered into the study. The patients had no history of previous anticancer chemotherapy or radiation therapy at the time of the study. The patients received an i.m. injection of a single dose of $2 \times 10^6$ international units of human lymphoblastoid IFN-$\alpha$ (Heriff Medical Aps, Omme, Denmark). The purity of the IFN preparation was $10^6$ IU of human IFN-$\alpha$ $mg^{-1}$ protein. Normal healthy donors were used as roughly age- and sex-matched controls.

### Cell preparation

Lymphocyte-rich mononuclear cells were isolated from heparinized peripheral blood by centrifugation on Ficoll–Hypaque gradients and further fractionated as described previously (Uchida & Micksche, 1981a, 1981b, 1983a, 1983b, 1983c). Mononuclear cells collected from the interface were washed and suspended in RPMI 1640 medium supplemented with 2 mM L-glutamine, 25 mM HEPES, 100 U penicillin $ml^{-1}$, 100 $\mu g$ streptomycin $ml^{-1}$, and 10% heat-inactivated foetal calf serum (FCS) (Gibco Bio-Cult, Glasgow, Scotland) (complete medium). The mononuclear cells were then incubated for 1 h at 37°C in plastic dishes that had been precoated with FCS. After incubation, nonadherent cells were removed, and the dish was washed with cold medium. Adherent cells were harvested from the dish after 15 min incubation with Versene (1/5000, Gibco Bio-Cult) and by vigorous washing with a pipette, then washed and suspended in complete medium. The adherent cells contained >94% monocytes as judged by morphologic examination and nonspecific esterase staining. The nonadherent cells were further incubated on nylon–wool columns for 1 h at 37°C, and then eluted with warm medium. The nylon–wool nonadherent cells contained >98% lymphocytes as judged by morphologic examination of Giemsa-stained smears. Every fraction was >97% viable by dye exclusion. Effector cells were used either immediately or stored at 4°C at a concentration of $10^6$ $ml^{-1}$ in complete medium, since under the condition there were no differences in cytotoxicity of fresh and cultured cells (Uchida & Micksche, 1981a).

### $^{51}Cr$ release cytotoxicity assay

A 4 h $^{51}Cr$ release assay was performed using the K562 human erythromyeloid leukemia cell line (Lozzio & Lozzio, 1975) as targets, as detailed elsewhere (Uchida & Micksche, 1981a, 1981b, 1983a, 1983b, 1983c). Briefly, 100 $\mu l$ $^{51}Cr$-labelled target cells ($5 \times 10^3$) and 100 $\mu l$ effector cells in different numbers were added to each well of round-bottomed microtiter plates. After 4 h incubation, the supernatant was collected, and the specific $^{51}Cr$ release in percentage cytotoxicity was calculated by the formula for triplicate samples: % Cytotoxicity = (test cpm − spontaneous cpm)/(maximum cpm − spontaneous cpm) × 100.

Lytic units (LU) were calculated from dose-response curves obtained by different numbers of effector cells by using linear regression analysis, as described previously (Uchida & Micksche, 1983b). One LU was defined as the number of effector cells required to induce 20% lysis of target cells, and the results are expressed as the number of LU/$10^6$ cells.

### Agarose single cell cytotoxicity assay

This assay was performed as described previously (Uchida & Micksche, 1983a; Uchida et al., 1984). Equal numbers ($2 \times 10^5$) of nylon–wool nonadherent lymphocytes and K562 were mixed in 0.2 ml medium, incubated for 10 min at 37°C, and centrifuged at 100 g for 5 min, followed by gentle suspension with a pipette. One per cent agarose (0.5 ml; Sea Plaque, Marine Colloids, Rockland, MA, USA) that had been kept in the liquid phase at 37°C was added to the conjugate suspension. One hundred $\mu l$ of the agarose-conjugate mixture were transferred on to agar-precoated microscope slides. After the solidification of agarose, the slide was placed in plastic dishes, filled with warm complete medium, and incubated for 4 h at 37°C. After incubation, the slide was stained with 0.2% trypan blue and fixed with 1% formaldehyde. The percentage of lymphocytes forming conjugates with K562 was determined by counting 200 lymphocytes, and that of lytic conjugate with dead target cells was scored by counting 100 conjugates. Spontaneous target cell death was assessed by counting 200 target cells in samples incubated in the absence of effector cells and did not exceed 5%. The percentage of active killer cells was calculated by the formula: % Active killer cells = % conjugate × % lytic conjugate × (1 − % spontaneous target death).

### In vitro *treatment with IFN or OK432*

Effector cells at a concentration of $10^6\,ml^{-1}$ in complete medium were incubated alone or with 1,000 IU IFN/ml (Heriff Medical Aps) or 50 μg OK432/ml (Chugai Pharmaceutical Co., Tokyo, Japan) for 12 h at 37°C in a humidified 5% $CO_2$ atmosphere, as described previously (Uchida & Micksche, 1981*b*, 1983*b*, 1983*c*). After incubation, the cells were washed and suspended in complete medium.

### *NK suppressor assay*

Effector cells ($10^6\,ml^{-1}$) in complete medium were precultured overnight alone or with half the number of cells used as suppressors either in the presence or absence of IFN (1,000 $IU\,ml^{-1}$), as described previously (Uchida & Micksche, 1981*a*, 1983*b*). After incubation the cells were harvested, washed and suspended in complete medium. There were no differences in the recovery of viable effector cells cultured alone and with suppressor cells.

### *Statistical analysis*

All determinations were made in triplicate, and the results were evaluated for statistical significance by Student's *t*-test.

### Results

### *Blood NK cell activity after IFN administration*

Peripheral blood lymphocytes (PBL) from cancer patients receiving IFN injection and from normal donors were tested for cytotoxicity against K562 in a 4 h $^{51}Cr$ release assay before and 2, 4, 12, and 24 h after a single injection of $2 \times 10^6$ IU IFN. Blood NK cell activity remained unchanged during the first 2 h (Table I). A depression of blood NK cell activity was observed 4 h after IFN injection and reached a nadir at 12 h. Blood NK cell activity then returned to and exceeded pretreatment levels within 24 h. In contrast, no significant changes in blood NK cell activity was found in normal controls during the observation period of 24 h. These results indicate that the change in blood NK cell activity observed in cancer patients treated with IFN is not attributed to the diurnal variation of blood NK cell activity nor due to time-related variance of cytotoxicity assays but results from the effects of injected IFN on the NK system of the patients. Results of 12 patients are shown in Figure 1. The initial decline in blood NK cell activity at 12 h points of IFN treatment was documented in 10/12 (83.3%) patients. Twenty-four hours after IFN injection blood NK cell activity returned to base line levels in 2 patients and exceeded pretreatment levels in another 8 patients. Thus, these patients were considered as responders. The other 2 patients, however, showed no depression nor increase in

**Table I** Kinetics of blood NK cell activity after IFN administration

| *Period after IFN injection (h)* | *IFN-treated*[a] | | *Control*[b] | |
|---|---|---|---|---|
| | *% Cytotoxicity*[c] | *LU/10*[6] | *% Cytotoxicity* | *LU/10*[6] |
| 0 | 32·4 | 41.9 | 42.9 | 83.6 |
| 2 | 31.5 | 40.0 | 41.4 | 80.0 |
| 4 | 24.7[d] | 28.1[d] | 44.6 | 86.9 |
| 12 | 6.2[d] | 4.8[d] | 43.5 | 81.4 |
| 24 | 40.6[d] | 74.1[d] | 42.3 | 80.5 |

[a]Blood lymphocytes from a cancer patient and a normal donors were tested for cytotoxicity against K562 in a 4 h $^{51}Cr$ release assay before and 2, 4, 12, and 24 h after IFN injection ($1 \times 10^6$ U) to the patients.
[b]No IFN given.
[c]At an effector to target cell ratio (E:T) of 10:1.
[d]Value is significantly different from that of 0 h ($P < 0.05$).

**Figure 1** Blood NK cell activity of patients before and 12 h and 24 h after IFN injection. Data are expressed as % cytotoxicity against K562 at an E:T of 10:1 in a 4 h $^{51}Cr$ release assay.

blood NK cell activity during 24 h post IFN administration and thus were considered as nonresponders.

On the other hand, there were no significant differences in the frequencies of LGL among PBL obtained before and 12 h and 24 h after IFN injection [14.3±1.8% *vs.* 13.5±1.7% *vs.* 14.6±1.8% (mean±s.e.)]. While the NK cells are not synonymous with or equivalent in number to LGL, more than 50% LGL have been reported to function as active NK cells against highly sensitive target cells (Timonen *et al.*, 1982). Taken together, these results suggest that the initial decline or the later increase in blood NK cell activity after IFN administration is unlikely to be derived from a redistribution of NK cells but may rather result from a reduction of the lytic activity of blood NK cells.

### *Analysis of NK cell activity at single cell level*

In an attempt to determine whether the initial decline in blood NK cell activity following IFN administration is attributable to a decrease in frequency of lymphocytes binding to target cells or due to a reduction of post-binding lytic function of effector cells, nylon–wool nonadherent cells were tested for their binding capacity and killing activity in a 4 h single cell cytotoxicity assay in agarose before and 12 h after IFN injection. The frequency of lymphocytes forming conjugates with K562 remained unchanged during the first 12 h of IFN treatment (Table II). There was no difference in the

**Table II** Reduction of NK cell activity after IFN administration determined in single cell cytotoxicity assay in agarose

| | *% Conjugate* | | *% Lytic conjugate* | | *% Active killer* | |
|---|---|---|---|---|---|---|
| *Patient* | *0 h* | *12 h* | *0 h* | *12 h* | *0 h* | *12 h* |
| 1 | 13 | 11 | 25 | 8[b] | 3.39 | 0.84[b] |
| 2 | 6 | 6 | 14 | 9[b] | 0.80 | 0.51[b] |
| 3 | 9 | 7 | 18 | 11[b] | 1.55 | 0.74[b] |
| 4 | 9 | 10 | 17 | 12[b] | 1.45 | 1.14[b] |
| 5 | 14 | 13 | 30 | 29 | 4.07 | 3.94 |
| 6 | 16 | 16 | 26 | 25 | 3.95 | 3.80 |
| 7 | 8 | 8 | 15 | 11[b] | 1.15 | 0.84[b] |
| 8 | 10 | 9 | 23 | 10[b] | 2.19 | 0.86[b] |
| 9 | 12 | 11 | 25 | 10[b] | 2.85 | 1.05[b] |
| 10 | 15 | 14 | 26 | 9[b] | 3.74 | 1.21[b] |
| 11 | 10 | 10 | 21 | 12[b] | 2.04 | 1.16[b] |
| 12 | 7 | 5 | 15 | 7[b] | 1.02 | 0.34[b] |

[a]Nylon–wool nonadherent lymphocytes obtained before (0 h) and 12 h after IFN injection were tested for binding and killing activities in a 4 h single cell cytotoxicity assay in agarose.

[b]Value is significantly lower than that of 0-h ($P < 0.05$).

mean percentage conjugates before and 12 h post IFN injection (9.9 ± 0.8% *vs.* 9.1 ± 0.8%). In contrast, the number of conjugates with dead target cells was reduced by IFN administration. The mean percentage of lytic conjugates observed 12 h post IFN injection was significantly lower than that observed before IFN injection (9.9 ± 0.5% *vs.* 19.9 ± 1.3%). Thus, the frequency of active NK cells among PBL obtained 12 h post IFN injection was estimated to be significantly lower than that before the initiation of IFN therapy (0.87% ± 0.08% *vs.* 2.02 ± 0.29%). The reduction of active NK cells was recorded for all 10 responder patients. On the other hand, nonresponder patients showed no changes in frequencies of conjugates, lytic conjugates, and active NK cells with IFN administration. These results indicate that IFN administration to cancer patients results in a rapid reduction of active NK cells in the peripheral blood and that this could be responsible for the initial decline in blood NK cell activity in the IFN-treated patients.

### In vitro *responsiveness to NK-enhancing effect of IFN of IFN-treated patients*

Since IFN has been reported to enhance the post-binding lytic function of effector cells (Ortaldo & Herberman, 1980; Targan & Dorey, 1980) and since the post-binding lytic activity was found to be impaired in PBL obtained from patients 12 h after IFN injection (Table II), PBL obtained 12 h post IFN injection were stimulated *in vitro* with the same IFN preparation that had been administred to the patients. *In vitro* exposure to IFN failed to augment NK cell activity both from responders and nonresponders (Figure 2B). Other types of IFN, IFN-β or IFN-γ, were also incapable of activating PBL obtained 12 h post IFN injection (data not shown). The unresponsiveness to *in vitro* IFN of PBL from responders was induced by IFN administration since PBL obtained before IFN injection were activated *in vitro* by IFN to express enhanced NK cell activity (Figure 2A). In contrast, no positive reactions were recorded for PBL from nonresponders regardless of IFN administration. These results indicate that IFN administration to cancer patients renders PBL unresponsive to *in vitro* NK-boosting effects of IFN by 12 h despite that these PBL retain their ability to bind target cells.

### *Recovery from unresponsiveness to IFN of PBL after* in vitro *culture*

The next set of experiments was performed to examine whether PBL isolated from patients 12 h after IFN administration could again obtain their responsiveness to IFN. Nylon–wool nonadherent lymphocytes obtained at 12 h points of IFN injection were cultured *in vitro* overnight in complete medium and then stimulated with IFN for 12 h. These cultured lymphocytes showed augmented NK cell activity in response to IFN, whereas fresh lymphocytes showed no such augmentation (Table III). There were no significant differences in cytotoxicity of fresh and cultured lymphocytes. On the other hand, nonadherent lymphocytes from nonresponder patients were still unresponsive to NK-enhancing effects of IFN even after *in vitro* culture (data not shown). These results indicate that PBL obtained from patients 12 h post IFN administration recover from unresponsiveness

**Figure 2** *In vitro* effects of IFN on NK cell activity of blood lymphocytes obtained before (a) and 12 h (b) and 24 h (c) after IFN injection. Blood lymphocytes were incubated for 12 h alone or with 1,000 IU IFN $ml^{-1}$, then washed and tested for cytotoxicity against K562 at an E:T of 10:1 in a 4 h $^{51}Cr$ release assay. (●) responder patients who showed augmented blood NK cell activity 24 h after IFN injection; (▲) responders whose blood NK cell activity returned to base line levels 24 h post IFN injection; (■) nonresponders whose blood NK cell activity was not modified by IFN injection.

**Table III** Recovery from unresponsiveness to IFN of blood lymphocytes obtained 12 h post IFN injection by *in vitro* culture

| | | *% Cytotoxicity*[b] | |
|---|---|---|---|
| *Patient* | *Effector cells*[a] | *Medium* | *IFN*[c] |
| 3 | Fresh | 8.9 | 8.3 |
| | Cultured | 8.7 | 21.1[d] |
| 4 | Fresh | 15.1 | 12.9 |
| | Cultured | 15.4 | 29.5[d] |
| 8 | Fresh | 11.3 | 11.2 |
| | Cultured | 14.0 | 26.8[d] |
| 9 | Fresh | 11.2 | 6.3 |
| | Cultured | 12.5 | 23.7[d] |
| 10 | Fresh | 16.7 | 14.7 |
| | Cultured | 19.6 | 33.7[d] |

[a]Nylon–wool nonadherent lymphocytes obtained 12 h after IFN injection were used either immediately or after overnight culture *in vitro* in complete medium.

[b]Cytotoxicity against K562 at an E:T of 10:1 in a 4 h $^{51}Cr$ release assay.

[c]Fresh or cultured lymphocytes were incubated alone or with $10^3$ U IFN ml$^{-1}$ for 12 h, then washed and tested.

[d]Value is significantly higher than that of corresponding control cells ($P<0.05$).

**Table IV** Suppression of IFN-induced augmentation of NK cell activity by monocytes obtained 12 h post IFN injection

| | *% Cytotoxicity of 0-h lymphocytes*[a] | | | |
|---|---|---|---|---|
| | *0-h monocytes added* | | *12-h monocytes added* | |
| *Patient* | *Medium* | *IFN* | *Medium* | *IFN* |
| 1 | 31.0 | 40.4[b] | 30.5 | 29.1 |
| 2 | 9.5 | 37.5[b] | 11.7 | 12.8 |
| 3 | 20.3 | 31.8[b] | 19.0 | 18.6 |
| 4 | 20.0 | 37.1[b] | 18.4 | 20.1 |
| 5 | 50.9 | 49.7 | 48.3 | 47.9 |
| 6 | 50.1 | 52.5 | 53.4 | 56.6 |
| 7 | 31.1 | 44.3[b] | 35.0 | 35.0 |
| 8 | 32.1 | 41.6[b] | 27.3 | 42.4[b] |
| 9 | 30.6 | 50.8[b] | 30.6 | 33.8 |
| 10 | 43.0 | 68.2[b] | 45.6 | 51.4 |
| 11 | 29.0 | 37.7[b] | 31.2 | 31.1 |
| 12 | 17.3 | 28.8[b] | 15.0 | 18.9 |

[a]Nylon–wool nonadherent cells obtained before IFN injection (0-h lymphocytes) were incubated for 12 h alone or with IFN ($10^3$ U ml$^{-1}$) either in the presence or absence of half the number of monocytes obtained before (0-h monocytes) or 12 h after (12-h monocytes) IFN injection, then washed and tested for cytotoxicity against K562 at an E:T of 10:1 in a 4 h $^{51}Cr$ release assay.

[b]Value is significantly higher than that of controls ($P<0.05$).

to IFN after *in vitro* culture and thus contain IFN-inducible pre-NK cells.

### *Suppression of IFN-induced augmentation of NK cell activity by monocytes obtained 12 h post IFN administration*

Adherent types of suppressor cells have been shown to inhibit the development of functional NK cells by IFN (Uchida & Micksche, 1983*b*; Uchida *et al.*, 1984*b*). To ascertain whether adherent cells obtained 12 h post IFN administration suppress IFN-induced enhancement of NK cell activity, nonadherent lymphocytes isolated from untreated patients were stimulated *in vitro* with IFN in the presence of blood monocytes obtained either before or 12 h after IFN injection. *In vitro* exposure to IFN caused an enhancement of NK cell activity in the presence of monocytes from untreated patients (Table IV). In the presence of monocytes obtained 12 h post IFN injection, however, nonadherent lymphocytes failed to express augmented NK cell activity in response to IFN, although the base line level of NK cell activity was not inhibited by these monocytes. The presence of suppressor monocytes was recorded in 9/10 (90.0%) responder patients 12 h post IFN injection. Nonadherent lymphocytes had no such suppressive activity (data not shown). These results suggest that IFN administration to cancer patients results in a rapid appearance of suppressor monocytes capable of inhibiting IFN-induced development of NK cell activity in the peripheral blood and that this could be involved in the rapid loss of reactivity to IFN of PBL observed in the patients 12 h after IFN injection.

### In vitro *augmentation of NK cell activity by OK432*

Since OK432 has been demonstrated to enhance NK cell activity in the presence of adherent suppressor cells (Uchida & Micksche, 1983*b*), attempts were next made to investigate whether NK cell activity of PBL obtained from patients 12 h post IFN administration is augmented by OK432 and whether OK432 enhances NK cell activity in the presence of suppressor monocytes from IFN-treated patients. *In vitro* treatment with OK432 resulted in an enhancement of NK cell activity of nonadherent lymphocytes obtained from untreated patients in the presence of suppressor monocytes obtained 12 h post IFN injection (Table V). Furthermore, PBL obtained from patients 12 h post IFN injection were activated by OK432 to show enhanced NK cell activity. These results indicate that the peripheral blood of cancer patients at 12 h points of IFN treatment contains cytotoxic

**Table V** *In vitro* augmentation of NK cell activity by OK432

| | | % Cytotoxicity[b] | |
|---|---|---|---|
| *Patient* | *Effector cells*[a] | *Medium* | *OK432* |
| 2 | 0-h lymphocytes | 10.9 | 46.4[c] |
| | 0-h lymphocytes + 12-h monocytes | 11.7 | 36.1[c] |
| | 12-h lymphocytes | 5.4 | 19.9[c] |
| 3 | 0-h lymphocytes | 22.4 | 38.6[c] |
| | 0-h lymphocytes + 12-h monocytes | 19.0 | 35.6[c] |
| | 12-h lymphocytes | 8.9 | 28.5[c] |
| 4 | 0-h lymphocytes | 22.1 | 35.6[c] |
| | 0-h lymphocytes + 12-h monocytes | 18.4 | 34.8[c] |
| | 12-h lymphocytes | 15.2 | 26.0[c] |
| 11 | 0-h lymphocytes | 30.3 | 60.0[c] |
| | 0-h lymphocytes + 12-h monocytes | 31.2 | 56.6[c] |
| | 12-h lymphocytes | 16.5 | 52.0[c] |
| 12 | 0-h lymphocytes | 12.8 | 31.0[c] |
| | 0-h lymphocytes + 12-h monocytes | 15.0 | 38.5[c] |
| | 12-h lymphocytes | 3.5 | 15.0[c] |

[a]Nylon–wool nonadherent lymphocytes obtained before IFN injection (0-h lymphocytes), 0-h lymphocytes plus half the number of monocytes obtained 12 h post IFN injection (12-h monocytes), and nonadherent lymphocytes obtained 12 h post IFN (12-h lymphocytes) were each incubated for 12 h alone or with OK432 (50 $\mu g\, ml^{-1}$), then washed and tested for cytotoxicity.

[b]Cytotoxicity against K562 at an E:T of 10:1 in a 4 h $^{51}Cr$ release assay.

[c]Value is significantly higher than that of controls ($P < 0.05$).

potential, which could be activated by OK432, but not by IFN.

*Reactivity of IFN of PBL obtained 24 h post IFN administration and possible role of suppressor monocytes*

It seemed important to test whether suppressor monocytes were still present in the peripheral blood of patients 24 h after IFN administration. First, PBL obtained from patients 24 h post IFN injection were stimulated *in vitro* with IFN. IFN failed to augment NK cell activity in 8/9 (88.9%) cases where blood NK cell activity was already enhanced *in vivo* by IFN injection at 24 h (Figure 2C). An increase in NK cell activity was recorded for one PBL sample whose NK cell activity did not exceed base line levels 24 h after IFN injection. PBL from two nonresponsers were still unresponsive to IFN. Next, PBL from normal donors were incubated alone or with IFN either in the presence or absence of blood monocytes obtained from patients 24 h after IFN administration. *In vitro* treatment with IFN resulted in an augmentation of NK cell activity of control lymphocytes regardless of the presence of monocytes (Table VI). These blood monocytes also

**Table VI** Effects of monocytes obtained 24 h post IFN injection on NK cell activity

| | | % Cytotoxicity[b] | |
|---|---|---|---|
| *Experiment* | *24-h monocytes added*[a] | *Medium* | *IFN* |
| 2 | None | 33.0 | 46.0[c] |
| | Added | 32.1 | 44.5[c] |
| 3 | None | 26.4 | 44.3[c] |
| | Added | 30.5 | 43.9[c] |
| 7 | None | 41.2 | 58.1[c] |
| | Added | 40.3 | 57.8[c] |
| 9 | None | 20.7 | 31.4[c] |
| | Added | 23.5 | 34.6[c] |
| 10 | None | 38.8 | 59.8[c] |
| | Added | 36.5 | 60.2[c] |

[a]Nylon–wool nonadherent cells from normal donors were incubated for 12 h alone or with IFN (1,000 $U\, ml^{-1}$) either in the presence or absence of half the number of monocytes from cancer patients (Nos. 2, 3, 7, 9, 10) obtained 24 h after IFN injection, then washed and tested.

[b]Cytotoxicity against K562 at an E:T of 10:1 in a 4 h $^{51}Cr$ release assay.

[c]Value is significantly higher than that of controls ($P < 0.05$).

had no suppressive activity to autologous blood lymphocytes obtained before IFN injection (data not shown). These results suggest that PBL obtained 24 h after IFN administration are activated *in vivo* by IFN to a great extent and therefore cannot respond *in vitro* to IFN and that blood monocytes are not involved in the unresponsiveness of these lymphocytes to NK-enhancing effects of IFN.

### Discussion

In the present report, several observations have been made concerning the effects of IFN on cytotoxic activity of NK cells and NK regulatory function of monocytes. A single i.m. injection of IFN-$\alpha$ to cancer patients has resulted in an initial decline in blood NK cell activity, being detectable at 4 h, reaching a nadir at 12 h and returning to or exceeding pretreatment levels within 24 h. Similar initial depression of blood NK cell activity after IFN administration has been documented in patients with chronic hepatitis at 4 h (Pape *et al.*, 1981), in normal donors at 6 h (Karimiemi *et al.*, 1980), and in cancer patients at 12 h (Koren *et al.*, 1983) or at 24 h (Golub *et al.*, 1982*a*, 1982*b*).

There are several possible explanations for the initial depression of blood NK cell activity caused by IFN administration; generation of suppressor elements, redistribution of NK cells, intrinsic defects in NK cells, and impaired development of active NK cells from pre-NK cells. Data presented in this report indicate that adherent blood cells obtained from patients 12 h after IFN injection are not suppressive to NK cells even after overnight contact. Nonadherent lymphocytes from IFN-treated patients also had no suppressor function (data not shown). Our findings are in keeping with previous reports that adherent suppressor cells are not involved in the initial decline in blood NK cell activity after IFN administration (Golub *et al.*, 1982*b*). In contrast, other investigators have reported that 2/3 patients develop nonadherent suppressor cells 12 h post IFN injection (Koren *et al.*, 1983). The reason for this discrepancy is not understood. It may be due to the differing responsiveness of patients' lymphocytes to IFN since the initial depression of blood KN cell activity after IFN administration is more profound in their patients than in ours.

It has been demonstrated that both pre-NK cells and active NK cells have the ability to recognize NK-susceptible target cells and that pre-NK cells lack the capacity to kill these target cells (Ortaldo & Herberman, 1980; Targan & Dorey, 1980; Reynold *et al.*, 1982). The single cell level assay in the present study has revealed that the frequency of lymphocytes forming conjugates with K562 target cells is not reduced at the time when blood NK cell activity is depressed in a population level assay. This may indicate that the total number of pre-NK cells and active NK cells is not affected by IFN administration. The single cell level assay has further demonstrated that the frequency of active NK cells is decreased along with the initial decline in blood NK cell activity determined in a $^{51}Cr$ release assay. In addition, the number of LGL among PBL has remained unchanged during the first 12 h of IFN treatment. Collective data suggest that the *in vivo* IFN-induced initial decline in blood NK cell activity is unlikely to be due to redistribution of NK cells following IFN administration but may rather result from suppression of functional development of active NK cells from pre-NK cells. Similar studies in another group have implied no change in the binding capacity of NK cells at the time of the initial depression of blood NK cell activity caused by IFN injection (Golub *et al.*, 1982*b*).

We have previously demonstrated that adherent cells from carcinomatous pleural effusions of cancer patients and from the peripheral blood of postoperative cancer patients suppress the maintenance of functional NK cells and development of active NK cells in response to IFN (Uchida & Micksche, 1981*a*, 1983*b*; Uchida *et al.*, 1982, 1984*a*). In the present study blood monocytes obtained from patients 12 h after IFN administration suppressed IFN-induced augmentation of NK cell activity, although these suppressor cells did not inhibit the maintenance of functional NK cells. The same type of adherent suppressor cells has quite recently been documented in bone marrow of normal individuals (Uchida *et al.*, 1984*b*). On the other hand, it has been proposed that both pre-NK cells and active NK cells are present in the peripheral blood and that endogenous IFN stimulates pre-NK cells to become active NK cells (Ortaldo & Herberman, 1980; Targan & Dorey, 1980; Reynold *et al.*, 1982). Taken together, it seems likely that the development of active NK cells from pre-NK cells is suppressed by blood monocytes in cancer patients shortly after IFN administration and that this could be responsible for the decline in blood NK cell activity in the patients observed 12 h post IFN injection. Indeed, blood NK cell activity is found to return to or exceed base line levels within 24 h of IFN therapy, and monocytes obtained at 24 h points of IFN treatment are no longer able to inhibit IFN-induced enhancement of NK cell activity. This suggests that blood monocytes rapidly lose their suppressive activity to NK cells *in vivo*. This suggestion was confirmed by *in vitro* data which showed that suppressor monocytes obtained 12 h post IFN injection lose their inhibitory capacity for IFN-dependent development of active NK cells after overnight culture *in vivo* (data not shown).

Previous reports have indicated that PBL obtained 12 h after IFN administration not only express depressed NK cell activity but also lack responsiveness to NK-boosting effects of IFN *in vitro* (Koren *et al.*, 1983). These authors argue that the initial decline in blood NK cell activity after IFN injection is in part due to a loss of specific cell type associated with NK cell activity. Similarly, PBL obtained 12 h post IFN injection failed to respond *in vitro* to IFN in the present study. However, the frequency of LGL among PBL and the number of lymphocytes forming conjugates with target cells were not reduced by IFN injection. Furthermore, PBL at 12 h points of IFN therapy recovered their responsiveness to NK-boosting effects of IFN after *in vitro* culture. In addition, PBL from IFN-treated patients were activated by OK432 to express increased NK cell activity. OK432 has been reported to enhance NK cell activity of LGL with pre-existing ability to recognize target cells (Uchida & Micksche, 1981*b*). Collectively, it seems likely that the initial depression of blood NK cell activity after IFN administration is unlikely to be due to a loss of NK cells in the blood but rather attributable to the unresponsiveness to IFN of pre-NK cells.

In conclusion, a significant but transient decline in blood NK cell activity has constantly been observed in cancer patients 12 h after IFN administration. The initial reduction of blood NK cell activity has been detected even after repeated injections of IFN (data not shown). The generation of suppressor monocytes capable of inhibiting IFN-dependent development of functional NK cells appears to be involved in the initial depression of blood NK cell activity, although the mechanism by which suppressor monocytes appear in the peripheral blood shortly after IFN injection remains unclear. It could be postulated that a transition of pre-NK cells to active NK cells is regulated by monocytes.

### References

BOLDIGNON, C., VILLA, F., ALLAVENA, P. & 4 others. (1982). Inhibition of natural killer activity by human bronchoalveolar macrophages. *J. Immunol.*, **129**, 587.

EINHORN, S., BLOMGREN, H. & STRANDER, H. (1980). Interferon and spontaneous cytotoxicity in man. V. Enhancement of spontanenous cytotoxicity in patients receiving human leukocyte interferon. *Int. J. Cancer*, **26**, 419.

EINHORN, S., BLOMGREN, H., STRANDER, H. & WASSERMAN, J. (1983). Influence of human interferon-α therapy on cytotoxic functions of blood lymphocytes. Studies on lectin-dependent cellular cytotoxicity, antibody-dependent cellular cytotoxicity, and natural killer cell activity. *Cancer Immunol. Immunother.*, **16**, 77.

GIDLUND, M., ORN, A., WIGZELL, J., SENIK, A. & GRESSER, I. (1978). Enhanced NK cell activity in mice injected with interferon and interferon inducers. *Nature*, **273**, 759.

GOLUB, S.H., DORSEY, F., HARA, D. & MORTON, D.L. (1982*a*). Systemic administration of human leukocyte interferon on melanoma patients. I. Effects on natural killer functions and cell populations. *J. Natl Cancer Inst.*, **68**, 703.

GOLUB, S.H., DOMORE, P. & RAINEY, M. (1982*b*). Systemic administration of human leukocyte interferon to melanoma patients. II, Cellular events associated with changes in natural cytotoxicity. *J. Natl Cancer Inst.*, **68**, 711.

GUTTERMAN, J.U., BLUMENSCHEIN, G.R., ALEXANIAN, R. & 9 others. (1980). Leukocyte interferon-induced tumor regression in human metastatic breast cancer, multiple myeloma, and malignant melanoma. *Ann. Intern. Med.*, **93**, 399.

HERBERMAN, R.B. (Ed.). (1980). *Natural Cell-Mediated Immunity Against Tumors*. Academic Press, New York, pp. **000–000**.

HERBERMAN, R.B. (Ed.). (1982). *NK Cells and Other Natural Effector Cells*. Academic Press, New York, pp. **000–000**.

HERBERMAN, R.B., ORTALDO, J.R. & BONNARD, G.Y. (1979). Augmentation by interferon of human natural and antibody-dependent cell-mediated cytotoxicity. *Nature*, **277**, 221.

HUDDLESTONE, J.R., MERIGRAN, Jr. T.C. & OLDSTONE, M.B.A. (1979). Induction and kinetics of natural killer cells in humans following interferon therapy. *Nature*, **282**, 417.

KARIMIEMI, A.L., TIMONEN, T. & KOUSA, M. (1980). Effect of leukocyte interferon on natural killer cells in healthy volunteers. *Scand. J. Immunol.*, **12**, 371.

KOREN, H.S., BRANDT, C.P., TSO, C.Y. & LASZLO, J. (1983). Modulation of natural killing activity by lymphoblastoid interferon in cancer patients. *J. Biol. Resp. Modif.*, **2**, 151.

LOTZOVA, E., SAVARY, C.A., GUTTERMAN, J.U. & HERSH, E.M. (1982). Modulation of natural killer cell-mediated cytotoxicity by partially purified and cloned interferon-α. *Cancer Res.*, **42**, 2480.

LOTZOVA, E., SAVARY, C.A., QUESADA, J.R., GUTTERMAN, J.U. & HERSH, E.M. (1983). Analysis of natural killer cell cytotoxicity of cancer patients treated with recombinant interferon. *J. Natl Cancer Inst.*, **71**, 903.

LOZZIO, C.B. & LOZZIO, B.B. (1975). Human chronic myelogenous leukemia cell line with positive Philadelphia chromosome. *Blood*, **45**, 321.

ORTALDO, J.R. & HERBERMAN, R.B. (1980). Characteristics of augmentation by interferon of cell-mediated cytotoxicity. In: *Natural Cell-Medicated Immunity Against Tumours*. (Ed. Herberman). New York: Academic Press, p. 593.

PAPE, G.R., HADAM, M.R., EIENBURG, J. & RIETMULLER, G. (1981). Kinetics of natural cytotoxicity in patients treated with human fibroblast interferon. *Cancer Immunol. Immunother.*, **11**, 1.

PRIESTMAN, T.J. (1980). Initial evaluation of human lymphoblastoid interferon in patients with advanced malignant disease. *Lancet*, I, 113.

REYNOLD, C.W., TIMONEN, T. & HERBERMAN, R.B. (1982). Pleiotrophic effects of interferon (IFN) on the augmentation of rat natural killer (NK) cell activity. In: *NK Cells and Other Natural Effector Cells* (Ed. Herberman). New York: Academic Press, p. 375.

SAKSELA, E., TIMONEN, T. & CANTELL, K. (1979). Human natural killer activity is augmented by interferon via recruitment of pre-NK cells. *Scand. J. Immunol.*, **10**, 257.

TARGAN, S. & DOREY, F. (1980). Interferon activation of "pre-spontaneous killer" (pre-SK) cells and alteration in kinetics of lysis of both "pre-SK" and active SK cells. *J. Immunol.*, **124**, 2157.

TIMONEN, T., ORTALDO, J.R. & HERBERMAN, R.B. (1981). Characterization of human large granular lymphocytes and relationship to natural killer and K cells. *J. Exp. Med.*, **153**, 569.

TIMONEN, T., ORTALDO, J.R. & HERBERMAN, R.B. (1982). Analysis by a single cell cytotoxicity assay of natural killer (NK) cell frequencies among human large granular lymphocytes and of the effects of interferon on their activity. *J. Immunol.*, **128**, 2514.

TRINCHIERI, G. & SANTOLI, D. (1978). Anti-viral activity induced by culturing lymphocytes with tumor-derived or virus-transformed cells. Enhancement of human natural killer cell activity by interferon and antagonistic inhibition of susceptibility of target cells to lysis. *J. Exp. Med.*, **147**, 1314.

UCHIDA, A., COLOT, M. & MICKSCHE, M. (1984*a*). Suppression of natural killer cell activity by adherent effusion cells of cancer patients: Suppression of motility, binding capacity and lethal hit of NK cells. *Br. J. Cancer*, **47**, 17.

UCHIDA, A., KOLB, R. & MICKSCHE, M. (1982). Generation of suppressor cells for natural killer activity in cancer patients after surgery. *J. Natl Cancer Inst.*, **68**, 735.

UCHIDA, A. & MICKSCHE, M. (1981*a*). Suppressor cells for natural killer activity in carcinomatous pleural effusions of cancer patients. *Cancer Immunol. Immunother.*, **11**, 255.

UCHIDA, A. & MICKSCHE, M. (1981*b*). *In vitro* augmentation of natural killing activity by OK432. *Int. J. Immunopharmacol.*, **4**, 365.

UCHIDA, A. & MICHSCHE, M. (1983*b*). Intrapleural administration of OK432 in cancer patients: Activation of NK cells and reduction of suppressor cells. *Int. J. Cancer*, **31**, 1.

UCHIDA, A. & MICKSCHE, M. (1983*a*). Lysis of fresh human tumor cells by autologous large granular lymphocytes from peripheral blood and pleural effusions. *Int. J. Cancer*, **32**, 37.

UCHIDA, A., YAGITA, M., SUGIYAMA, H., HOSHINO, T. & MOORE, M. (1984*b*). Strong natural killer (NK) cell activity in bone marrow of myeloma patients: Accelerated maturation of bone marrow NK cells and their interaction with other bone marrow cells. *Int. J. Cancer*, **34** (in press).